# Characteristics of the Gut Bacterial Composition in People of Different Nationalities and Religions

**DOI:** 10.3390/microorganisms10091866

**Published:** 2022-09-18

**Authors:** Mikhail Syromyatnikov, Ekaterina Nesterova, Maria Gladkikh, Yuliya Smirnova, Mariya Gryaznova, Vasily Popov

**Affiliations:** 1 Laboratory of Metagenomics and Food Biotechnology, Voronezh State University of Engineering Technologies, 394036 Voronezh, Russia; 2 Department of Genetics, Cytology and Bioengineering, Voronezh State University, 394018 Voronezh, Russia

**Keywords:** gut, microbiota, nationalities, religion, diseases

## Abstract

High-throughput sequencing has made it possible to extensively study the human gut microbiota. The links between the human gut microbiome and ethnicity, religion, and race remain rather poorly understood. In this review, data on the relationship between gut microbiota composition and the nationality of people and their religion were generalized. The unique gut microbiome of a healthy European (including Slavic nationality) is characterized by the dominance of the phyla *Firmicutes*, *Bacteroidota*, *Actinobacteria*, *Proteobacteria*, *Fusobacteria*, and *Verrucomicrobia*. Among the African population, the typical members of the microbiota are *Bacteroides* and *Prevotella*. The gut microbiome of Asians is very diverse and rich in members of the genera *Prevotella, Bacteroides Lactobacillus, Faecalibacterium*, *Ruminococcus*, *Subdoligranulum*, *Coprococcus*, *Collinsella*, *Megasphaera*, *Bifidobacterium*, and *Phascolarctobacterium*. Among Buddhists and Muslims, the *Prevotella* enterotype is characteristic of the gut microbiome, while other representatives of religions, including Christians, have the *Bacteroides* enterotype. Most likely, the gut microbiota of people of different nationalities and religions are influenced by food preferences. The review also considers the influences of pathologies such as obesity, Crohn’s disease, cancer, diabetes, etc., on the bacterial composition of the guts of people of different nationalities.

## 1. Introduction

The gut microbiota is a complex and dynamic community of microorganisms, the unique composition of which is specific to each person. Microbiome formation takes place over several years of life from the moment of birth [1,2,3,4]. The number of bacteria inhabiting the intestines, on average, reaches about one hundred trillion [5]. In total, about 800 species live in the human intestine, and this microbial community weighs ~1–2 kg. The main members of the gut microbiota are *Bacteroidota*, *Firmicutes*, *Proteobacteria*, *Actinobacteria*, *Fusobacteria*, *Verrucomicrobia*, and *Cyanobacteria*, and they are represented in different proportions [6]. Together, these microorganisms have a direct effect on the functioning of the body, affecting metabolic processes, physiology, and human immunity, as well as maintaining homeostasis and influencing changes in the bacterial composition, whose disturbance can contribute to the development of various metabolic diseases [7,8].

In recent decades, the scientific community has been actively conducting research aimed at studying the microbiome and the factors that affect its composition [9]. However, it should be noted that, presently, there are no generally accepted definitions of a healthy human microbiome, its bacterial composition, and the ratio of microorganisms within it. To resolve this issue, it is necessary to improve the microbiome profiling methods that enable strain identification [10]. Numerous studies have been devoted to the development of diseases of various origins and the role of the intestinal microbiome in these processes [11,12,13,14,15]. The topics related to the progression of processes in the body [16,17,18,19,20,21], as well as the relationship between the gut microbiome and obesity, remain especially relevant [22,23,24,25]. Of great importance is the research aimed at studying the development of, and changes in, the microbiota with age, as a result of a particular diet, and under the influence of various environmental factors [26,27,28,29].

There are a very limited number of reviews that consider the links between the human gut microbiome, ethnicity, and race [30,31,32]. These reviews focus more on the influences of geographic and environmental factors on the human microbiome. Very little attention is paid specifically to the ethnicity of a person. We did not find any reviews on the relationship between gut microbiota and religion. This topic is of interest since the microbiomes of such groups are formed under the influence of many factors that are specific and, in some cases, unique (geography, genetics, lifestyle, diet, religion, etc.). In this regard, this work is devoted to the review of scientific research on the bacterial composition of the intestines of various ethnic groups, races, and nationalities.

## 2. Nationality and Gut Microbiota

The change in the composition of the human gut microbiome is not only determined by the state of health. The criteria also include racial and ethnic differences [33]. Publications of scientific works devoted to this area are quite rare due to the complexity of the structure of the study (difficulties in repeating generalizations) [30]. However, despite this, researchers are increasingly studying the issues of differences in the gut microbiome across populations.

The formation of bacterial communities in the gut does not only involve genetic potential [34]. The process of the formation of the gut microbiota can be characterized as multifactorial. It includes the influences of psychological health, socio-cultural environment, diet, lifestyle, and many other factors [30,35,36,37,38]. All these factors, together, contribute to the formation of a unique gut bacterial profile. Historical lifestyles and diet are among the main criteria shaping racial and ethnic differences in the microbiome. The composition of the gut can differ not only between urban and rural populations but also within the same community, which is explained by differences in ethnicity [39,40,41].

In 2014, the gut microbiome of Hadza men and women was analyzed. Based on the data obtained, a relationship between the bacterial diversity of the gut and gender, as well as the division of labor, was shown. There was an increased content of *Treponema* in the intestines of women and a predominance of *Eubacterium* and *Blautia* in men [34].

The study of intestinal bacterial diversity depending on ethnicity has been most actively investigated in Asian countries, where the subjects of the study were adults and children, healthy people, and people with various diseases [41,42,43,44]. Thus, the microbiomes of four Malaysian communities were studied, including Malays, the Chinese population, Indians, and one of the indigenous tribes, the Jakun [41]. *Prevotella, Bacteroides*, and *Bifidobacterium* were the dominant taxa. The Indigenous population and the Chinese population were characterized by a high proportion of *Prevotella* and *Bacteroides*, while a feature of the Jakun gut was the detection of *Klebsiella quasipneumoniae*. Probably, this is due to the tribe’s low access to clean water relative to the other cohorts. A large-scale study of the gut microbiome among the Japanese population was published in 2022 [44]. The objects of the study were 1803 people, including healthy people and people with diseases of various genesis. It was found that the Japanese population is characterized by the predominance of bacteria of genera such as *Bacteroides*, *Faecalibacterium, Blautia, Ruminococcus, Roseburia*, and *Prevotella* in the gut microbiome. The characteristic features of the Japanese microbiome were registered due to the discovery of an enterotype (type D) enriched with bacteria of the genus *Bifidobacterium*. In 2020, Jonguk Park and colleagues also conducted a large-scale study of the bacterial composition of the intestines of 1596 Japanese people. It was confirmed that a special feature of the Japanese microbiome is the high abundance of *Bifidobacterium* and *Blautia*.

The influences of the socio-economic aspects on the lives of children of different populations living in North Malaysia were investigated [40]. The study involved three groups of children, including Malays and the Chinese and Indigenous populations of Malaysia. It was found that the diversity of the gut microbiota was most pronounced in children from low-income families, who were aborigines.

A comparison of the gut microbiomes of students from India and China was carried out [45]. Indians were dominated by the two genera *Prevotella* and *Lactobacillus*, probably due to the high consumption of wheat by Indians, in contrast to the Chinese, whose diet is enriched with protein and animal fats. Many factors influencing the formation of the microbiome were considered, including the methods of childbirth and feeding [43,44,45,46]. In 2020, a study was conducted on the microbiota of newborns living within the same geographical area of Singapore [43]. The above factors were decisive in the formation of the ethnic characteristics of microbiomes. Increased levels of *Bifidobacterium* and *Lactobacillus* were characteristic of Indian infants, while Chinese children were characterized by high levels of *Bacteroides* and *Akkermansia*.

The results of the study of the Hong Kong and Yuan populations showed a high relative abundance of the *Actinobacteria* phylum in the Hong Kong population compared to the studied Yuan populations, which indicates that geography and ethnicity contribute, to varying extents, to the bacterial microbiome of the human intestine [47].

Racial and ethnic lifestyle characteristics have the greatest influence on the formation of differences in the microbiome composition between people [33,48]. For example, the analysis of the microbiomes of about two thousand residents of Amsterdam belonging to different ethnic groups, including the Dutch, Ghanaians, Moroccans, Turks, Africans, and South Asian Surinamese individuals, was performed. It turned out that the abundance of *Bacteroides*, *Firmicutes*, *Prevotella*, and *Clostridium* was one of the main factors determining the ethnic composition of the microbiome. Thus, among the Surinamese, *Bacteroides* was the dominant taxon, while the abundance of *Prevotella* was reduced. For the Moroccan and Turkish groups, the results were inverted due to their diets (increased fiber intake) [49].

Recently, another study was conducted on the influence of ethnicity on the structural diversity of microbiomes. The objects of the study were immigrants from East Asia and people of the Caucasian race living in the territories near San Francisco Bay [50]. In addition to nationality, the participants’ weight indicators played an important role in the experiment. According to the results of the study, it was found that bacterial differences were most pronounced in people with a lean body structure in both these two ethnic groups. Thus, *Akkermansia muciniphila, Bacteroidales bacterium ph8*, and *Roseburia hominis* were the main taxa of bacteria dominating the content of the guts of Caucasians. In turn, the microbiome of East Asian members of the population was characterized by an increased content of *Ruminococcus gnavus*.

**Figure 1:**
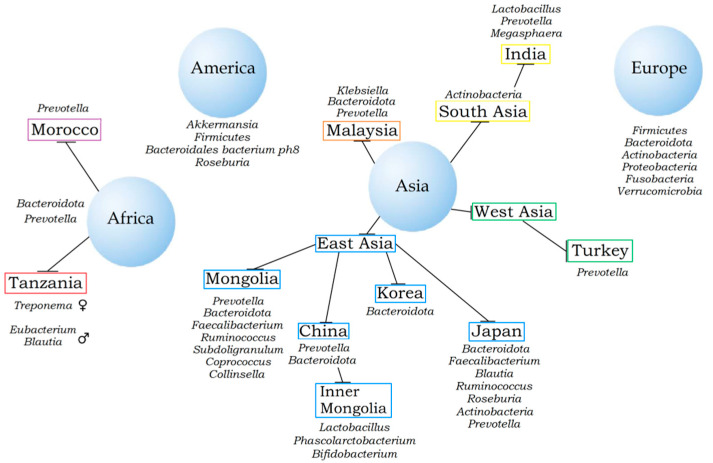
Features of the gut microbiota of different regional populations.

The African microbiome was also investigated [51,52]. The bacterial composition of the African cohort was enriched with *Bacteroides* and *Prevotella*, of which the latter facilitates the hydrolysis of cellulose and xylan. The presence of members of these genera in the microbiomes of the European cohort was not discovered. Morton and colleagues analyzed the impact of occupation (farmers and fishermen) on the composition of the gut microbiome in rural Cameroon [52]. It was found that the segment of the population who engaged in fishing has a much lower alpha diversity than farmers, which is associated not only with the geography of residence but also with the diet. This fact is confirmed by several studies that describe the dominance of certain taxa depending on food habits [53,54,55]. The abundance of *Prevotella* in the gut is associated with the predominance of plant foods in the diet since it improves the digestion of fibrous plant foods, and the increased content of *Bacteroides* is associated with the so-called “Western” diet, enriched with fats, proteins, and sugars [56]. This diet is typical of the inhabitants of America and Europe. The populations of China and neighboring Asian countries are characterized by an “oriental” diet (rice, noodles, soups, vegetables, and meat). Food preparation, drinks, and eating rituals also contribute to microbiota formation [56]. Young-Do Nam and colleagues analyzed the composition of the gut microbiome in the populations of Korea, Japan, and America. *Firmicutes* formed the dominant taxa among the Americans, *Actinobacteria* among the Japanese, and *Bacteroidota* among Koreans [57,58]. The uniqueness of the Japanese microbiome has been described in numerous articles [58,59,60,61]. The features of the habitat, diet, and genotype form the unique composition of the gut microbiota of the Mongolian population [61,62,63]. Members of the genera *Prevotella*, *Bacteroides*, *Faecalibacterium*, *Ruminococcus*, *Subdoligranulum*, and *Coprococcus* were found to be characteristic components of the microbiome of the Mongolian population [61]. Another study confirmed these findings and linked the abundance of *Collinsella* bacteria to a protein-rich diet [62]. Jing Li and colleagues conducted an experiment based on changing the Mongolian diet to a carbohydrate-rich diet [63]. The results obtained indicated a decrease in bacterial diversity in connection with the changes to a new diet. A similar experiment was also carried out by this research group in the territory of Inner Mongolia in China. This change in the diet contributes not only to a change in the composition of the bacteria but also to their metabolic pathways [64]. Differences in the microbiome composition of the intestine in the population of Inner Mongolia were also shown based on a comparison of two groups, including urban and rural residents [39]. For example, *Lactobacillus helveticus* was predominantly found in the intestines of rural residents. Features of the gut microbiomes of Mongolians, as well as members of other Asian, African, American, and European countries, are shown in Figure 1.

In 2016, Lei Chen conducted a large-scale study aimed at studying the expression of genes that can reliably distinguish between races (including people of Asian, European, and American descent) according to the microbiome composition, formed by dietary habits, living conditions, and metabolic rates [65]. As a result, specific genes that are characteristic of a particular race were identified. For example, the functional ppsA gene is specifically expressed only in *Pseudomonas stutzeri*, found in the guts of Europeans. AhpC is a unique gene of the Asian gut microbiome that is expressed in *Helicobacterium*, which explains the widespread occurrence of gastritis in Asian countries. As for the microbiome of the American population, the gene TVAG_129840-*Trichomonas vaginalis strain G3* is characteristic of it. Based on the works of various authors, it was found that the composition of the gut microbiome of the Indian population has features that distinguish Indian people from representatives of other ethnic groups and races [66,67,68]. It was found that the increased content of bacteria of the genus *Prevotella* and the genus *Megasphaera* is a hallmark of the Indian microbiome [67]. However, it has also been found that there are differences in bacterial composition among the ethnic tribes of India [69]. In 2019, Dhakan showed that the intestines of the population of north-central India, which have a predominantly plant-based diet, are enriched with bacteria of the genus *Prevotella*. By comparison, the dietary diversity of the South Indian cohort contributes to the dominance of members of the genera *Bacteroides*, *Ruminococcus*, and *Faecalibacterium* [68].

Alpha diversity is the intragroup diversity of the microbiota, and beta diversity is the bacterial diversity of the gut varying between different communities [33]. The study of the gut microbiome often relies on the analysis of alpha and beta diversity [33,70]. There were significant differences in alpha diversity among schoolchildren living in one of the cities of China (Han, Tibetan, and Hui populations) [70].

## 3. Gut Microbiota and Diseases of People of Different Nationalities

The study of the composition of the microbiome of various ethnic and racial groups is interesting in the context of its connection with many diseases, such as autism, cancer, irritable bowel syndrome, obesity, etc. [30,35,71,72,73,74,75]. In this regard, interdisciplinary collaboration is required. There is already a pool of studies linking ethnic microbiomes and health [30,35]. For example, members of the genus *Odoribacter*, which are primary butyrate producers, in the gut have been negatively associated with the severe consequences of Crohn’s disease. In this vein, residents of the Asia-Pacific Islands were found to be more likely to be hospitalized with the increased severity of the disease compared to the population of Latin America [35].

It has been suggested that the microbiota is associated with blood pressure, based on the level of metabolites, and the development of irritable bowel syndrome [75,76]. Members of the genus *Roseburia*, *Clostridium*, and *Romboutsia* were noted as the main bacteria influencing systolic blood pressure [75].

It is known that intestinal permeability is a risk factor for type-1 and type-2 diabetes [77,78,79]. Changes in the composition of the gut microbiome can also trigger the development of diabetes. A study of Turkish children was carried out at Istanbul University. The study found that an increase in the bacteria of the *Enterobacteriaceae* family may be a trigger of diabetes. The change in the bacterial ratio of the gut can also be used as a measure for the prevention and early detection of type-2 diabetes [80]. A group of Iranians with type-2 diabetes were characterized by increased levels of *Lactobacillus*, *Escherichia coli*, and *Bacteroides fragilis.* In turn, the pre-diabetic group were characterized by an increase in the content of *Escherichia coli* and *Bacteroides fragilis* in the gut in contrast to the composition of the guts of healthy people [81]. Changes in the composition of the gut microbiome with type-2 diabetes among representatives of two ethnic groups living in the same territory, the African and South Asian Surinamese populations, were studied [82]. Each of the two groups was divided into three subgroups, including healthy people, patients who were taking metformin, and patients who were not taking the drug. It was found that significant changes in the microbiome were observed in diabetic patients receiving therapy. This difference was most pronounced in the group of South Asian Surinamese individuals.

Racial differences in the microbiome are also linked to the development of colorectal cancer [71,72,83]. In 2018, two research teams analyzed the microbiomes of Afro-American and white-skinned people with colorectal cancer independently. A relationship was found between the severity of the disease and the ethnicity of the gut. The beta diversity composition was found to vary significantly between the studied groups of people; the dominance of *Faecalibacterium* and *Bacteroides* in the guts of Afro-Americans stood in contrast to the gut compositions of white-skinned people [72]. In 2022, a review was published on bacterial changes in the gut microbiome among patients with colorectal cancer, depending on ethnicity and race. Thus, after analyzing a large amount of experimental data, the authors concluded that patients with colorectal cancer are characterized by an increase in the levels of the genera *Bacteroides* and *Prevotella* and the species *Escherichia coli*, *Streptococcus gallolyticus*, *Enterococcus faecalis*, *Fusobacterium nucleatum*, and *Clostridium difficile* [83]. New aspects of the relationship between gut bacterial composition and the development of stomach cancer in the Mongolian population were discovered. It was found that bacteria of the genera *Enterococcus*, *Lactobacillus*, *Carnobacterium*, *Glutamicibacter*, *Paeniglutamicibacter*, *Fusobacterium*, and *Parvimonas* can act as tumor markers [84].

**Figure 2:**
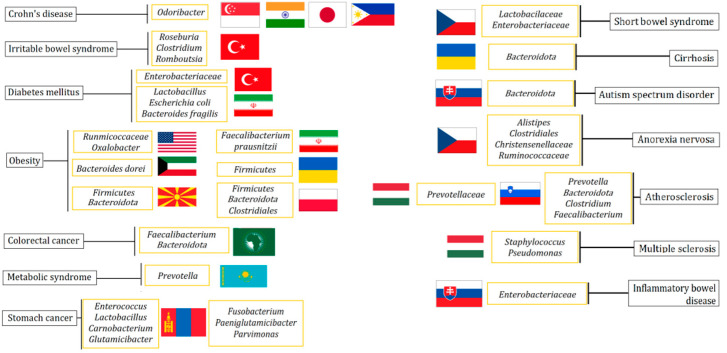
Bacterial taxa are typical of residents of different countries, the composition of which predominates during the development of the diseases.

The compositions of the intestinal microbiota of healthy citizens of Kazakhstan and people with metabolic syndrome were also compared in 2018. The data obtained were compared with the information available on the gut microbiomes of other nationalities and races, which made it possible to detect the features of the Kazakh cohort [85]. The diet and lifestyle of Kazakhs contributed to the formation of the *Prevotella* enterotype in their guts. The main difference between a healthy cohort of citizens of Kazakhstan and those diagnosed with metabolic syndrome was a decrease in the ratio of *Firmicutes*/*Bacteroidota*, as well as that of *Bifidobacterium*/*Subdoligranulum*, which was accompanied by an increase in the relative content of *Prevotella*. The connection between obesity and the composition of the microbiome was also studied in a racial-ethnic aspect [74,86,87,88]. Thus, two groups of children were studied, including European Americans and Afro-Americans. Among the Afro-Americans, two taxa, *Ruminococcaceae* and *Oxalobacter*, were found to be associated with obesity. The obese European Americans did not have disease-suggestive gut microbiota features. However, the differences between healthy and obese children within this group included the presence of *Aggregatibacter* and *Eikenella* in the oral microbiome of obese children [74]. Similar studies have also been conducted on adult populations in Arab countries [86,87,88]. Thus, in one of the studies, it was determined that all participants in the experiment showed a predominance of the bacteria of the *Bacteroides dorei* species. This can be connected with dietary habits since the majority of the Kuwaiti population is overweight [83]. Moreover, in a study of the Iranian population, it was found that the number of *Faecalibacterium prausnitzii* was increased in direct proportion to the increase in body weight. This fact can be used as a marker for the control and treatment of obesity [87]. Figure 2 presents a generalization of the experimental data on the increase in the content of a particular bacterial taxon during the development of diseases among representatives of various nationalities.

## 4. Religion and Gut Microbiota

Some studies have linked the composition of the gut microbiome to religion [89,90,91,92,93,94]. However, this connection is based on the diets followed by people of one religion or another [95]. Thus, the concept of Ayurveda, for many millennia, has continued to be based on the peculiarity of including specific foods in the diet (*Phyllanthus emblica*, *Terminalia bellerica*, and others). Diet affects the gut microbiota, which in turn can have a positive or negative effect on human health. A comparison of the composition of microbiomes between a control group and women adhering to meditation and veganism was performed. For the group practising meditation, the content of phyla such as *Actinobacteria* and *Proteobacteria*, as well as the genus *Bifidobacterium*, was reduced compared to the control group, who had never practised veganism or meditation [94,96]. At the same time, the intestines of the meditators were significantly enriched with *Subdoligranulum variabile*, *Roseburia hominis*, and *Butyricicoccus pullicaecorum* [94]. Chauhan and colleagues investigated the dependence of the microbiome composition on the endophenotype (Vata, Pitta, Kapha) of the individual [89]. Thus, women belonging to the Pitt type were characterized by the presence of members of the genus *Blautia* in the gut microbiome, an overabundance of *Butyricicoccus pullicaecorum*, and a specific enrichment of *Gemmiger formicilis*. The Kapha type group was characterized by the increased content of the bacteria *Prevotella copri*, and the Vata type was characterized by the abundance of *Phocaeicola vulgatus* and *Oscillibacter valericigenes*. Similar studies were conducted on a group of vegetarians and non-vegetarians. It was found that the majority of vegetarians were characterized by the presence of specific species, including *Clostridium nexile, Lachnospira eligens*, and *Prevotella copri*, in the gut. In contrast to the control group, which had an abundance of various pathogens (*Bilophila wadsworthia*, *E. coli*, and *E. hermannii*), *Klebsiella pneumonia* was the only species detected in the vegetarian group which, along with a high number of *P. copri*, can contribute to better health [97].

Seventh-day Adventists are a large cohort of people, a high percentage of whom are vegans or vegetarians [98]. Their diet is based mostly on the consumption of whole grains, legumes, nuts, and seeds [99]. For this reason, the gut microbiomes of members of Seventh-day Adventists are interesting objects of research on the influence of a vegetarian diet on the development of various diseases and certain health indicators, including body mass index, hypertension, obesity, diabetes, etc. [100,101]. A vegetarian diet has a positive effect on the lipid profile of the blood and also reduces the risk of coronary heart disease and diabetes [102]. It was found that a vegan, plant-based diet helps to reduce the likelihood of developing all types of cancer by 16% compared with non-vegans [99]. In addition, the analysis of the body mass index values among vegans showed lower values in contrast to vegetarians and omnivores, which may indicate the formation of a more varied intestinal microbiome amongst them [103]. Studies aimed at directly studying the taxonomic composition of the microbiome among the group of Seventh-day Adventists have not yet been published.

Ramadan is a religious practice, the essence of which is, in a sense, intermittent fasting. For one month, Muslims eat between sunset and sunrise 104]. As a result, the fasting microbiome is characterized by several features [90,91,92,93]. At the end of Ramadan, the gut community was shown to be enriched with the following bacterial taxa: *Butyricicoccus, Bacteroides, Faecalibacterium, Roseburia, Allobaculum, Eubacterium, Dialister*, and *Erysipelotrichia*. At the same time, the content of *Butyricicoccus pullicaecorum* was reduced relative to its concentration before the start of fasting [91,92,93]. The results of these studies are based on the fact that this type of fasting leads to an increase in the gut contents of *Akkermansia muciniphila* [91,92] and *Bacteroides fragilis* [91]. These bacteria are components of healthy gut microbiota, which is indicative of the beneficial effects of Ramadan on health.

It has also been found that fasting promotes a change in the beta diversity of the gut and some taxa [104]. The study of the microbiome of Pakistani and Chinese groups during Ramadan showed bacterial differences in the gut among different ethnic groups of Muslims. *Firmicutes* and *Actinobacteria* were dominant in pre-fasting Pakistanis compared to the Chinese, who showed an increase in *Bacteroidota*. At the end of the fast, the Chinese group showed increased levels of *Dorea, Klebsiella*, and *Faecalibacterium*. The Pakistani group was characterized by enrichment with *Sutterella*, *Parabacteroides*, and *Alistipes* [104]. Similar studies were performed by Li and colleagues [90]. A group of Han Chinese individuals and two groups of Muslims, including Kazakhs and Uyghurs, were selected as the objects of research. For the latter, the *Prevotella* type was the characteristic enterotype of the intestinal microbiome. The *Bacteroides* type was characteristic of the Chinese microbiota. Based on these studies, a conclusion was drawn regarding the correspondence between enterotypes and religions; i.e., for Buddhists and Muslims, the *Prevotella* type is characteristic, while for representatives of other religions, the *Bacteroides* type is characteristic [105].

## 5. Gut Microbiota of Slavic Peoples with and without Various Diseases

The study of the microbiome of the Slavs is of great scientific interest. This is due to the fact that representatives of this ethno-linguistic group are widely settled in the territories of Southern, Northern, and Eastern Europe. This geographical factor contributes to the features of culture and lifestyle, including nutrition, which is directly related to the characteristics of the human microbiome [106]. Scientific papers devoted to the study of gut bacterial diversity have been frequently published in the last decade. However, the study of the microbiome of Slavic groups remains a relevant direction for further active research, since many cohorts of Slavs have not been fully identified, and a number of them have not been studied [106,107,108,109,110].

The bacterial composition of the gut is directly dependent on lifestyle, diet, and diseases of various origins, including obesity, diabetes mellitus, pyelonephritis, hypertension, etc. [111,112,113]. The gut microbiota is associated with the human brain and other organ systems and thus affects the physiological processes in the body and its health [108].

Studies of the microbiome of the Slavs living in Western Europe, in countries such as Poland, the Czech Republic, Slovakia, and Hungary; in Southern Europe, including Slovenia and Macedonia; and in Eastern Europe, namely in Ukraine, mainly aimed to investigate the bacterial composition in regard to the pathological processes in the body and the norms [109,114,115,116].

It is important to note that studies of the gut microbiomes of healthy people are rarely found in scientific journals. However, a few studies that have been presented to date play a significant role in understanding the relationship between the human microbiome and factors of the external and internal environment [117]. The main bacteria that dominate the intestines of a healthy person are *Firmicutes*, *Bacteroidota*, *Actinobacteria*, *Proteobacteria*, *Fusobacteria*, and *Verrucomicrobia*. At the same time, about 90% of the gut microbiota are *Firmicutes* and *Bacteroidota* [118].

It is known that the dietary habits of people often directly affect the composition of the gut microbiome. For example, it has been suggested that the features of the microbiome composition of the Ukrainians may be associated with the consumption of rye bread and pork fat [119]. The dependence of the composition of the microbiota on seasonal changes, which are also associated with nutrition, was also traced [120]. Most of the population of Ukraine is mainly engaged in agriculture, which is directly related to the seasons. Therefore, some food products (fruit and vegetables) are not included in the diet of residents throughout the year. According to the study, *Actinobacteria* were the most abundant members of the microbiome in summer, and *Bacteroidota* were much less common in the samples, while the content of *Firmicutes* did not depend on the season.

The microbiome of Slovenians was also studied [121]. It is known that age, gender, and medications, as well as stress and dietary changes, can potentially alter the composition of the microbiota [122]. It was found that the diversity of bacteria in the gut increases with age. In addition, the diversity of the gut microbiota of Slovenian women was broader than that of men. At the same time, members of the genera *Faecalibacterium, Bacteroides*, and *Roseburia* were regular components of the microbiomes of all research objects [121]. Studies of this kind were carried out in Ukraine as well. It was shown that the contents of *Actinobacteria* and *Firmicutes* in the intestine increased, while *Bacteroidota* steadily decreased, with age. An increase in the *Firmicutes*/*Bacteroidota* ratio was correlated with age [119]. The developmental features of the microbiomes of Slovenian newborns were also described. The influences of factors such as childbirth, breastfeeding, and even the location of the maternity hospital on the formation of a stable microbiota in infants were revealed. For example, the share of *Enterococcus faecalis* in the microbiome depended on the mode of delivery, and the *Bacteroides*/*Prevotella* ratio was correlated with birth weight and the baby’s weight at the end of the first month of life [123].

One of the goals of studying gut microbiota is to discover the role of the microbiome in the development of diseases of various origins, including obesity. Currently, the gut microbiota is perceived by the scientific community as a separate “metabolic organ” [5,119]. Thus, the relationship between the gut bacterial composition and the body mass index of the adult population of Ukraine was revealed. A high concentration of *Firmicutes* and a lower level of *Bacteroidota* were found in obese people [119].

Recent studies have shown that gut bacteria play an important role in maintaining normal body weight. For more than a decade, a significant increase in the prevalence of obesity in adults and children has been recorded [124,125]. Poland is no exception in this regard. Gózd-Barszczewska, in 2019, studied the microbial composition of the intestines of obese middle-aged men who were prone to overweight and obesity. This study was the first attempt to analyze the gut microbiome in local communities. *Firmicutes* and *Bacteroidota* were found to be the predominant components of the microbiome, *Clostridia* and *Bacteroidia* were also common classes, and *Clostridiales* and *Bacteroidales* were the most numerous orders inhabiting the intestine [124]. Similar studies were carried out in Macedonia [126]. There was an increase in the *Firmicutes*/*Bacteroidota* ratio in the gut microbiota of obese people, specifically.

Work by Czech researchers was devoted to the study of the microbiome in short bowel syndrome, which described the effects of the intestinal microbiome on clinical outcomes of patients with this disease [109]. A characteristic feature of the intestines of such patients was a high abundance of *Lactobacillaceae* and *Enterobacteriaceae*.

It has been proved that the bacterial composition of the gut directly depends on the state of human health [107,127]. According to Polish scientists, using the example of liver cirrhosis of various origins, the microbiota can both improve and aggravate the course of the disease through various mechanisms [107]. A similar study of the influence of liver pathology on the features of the intestinal microbiome was conducted in Ukraine, in which there was an increase in the concentration of *Bacteroidetes* (by 37.11% and 21.30%, respectively), coupled with a decrease in *Firmicutes* (by 7.38% and 7.77%, respectively) [127].

As noted earlier, the composition of the gut microbiome is associated with the functioning of the brain [128]. The association of microbiome diversity with behaviour in autism spectrum disorders was described. According to this research, a high abundance of Lactobacillus supports the theory of dysbiosis in autism. Previously, similar results had been presented confirming the increase in the number of *Lactobacillus* in people with autism [129]. In addition, the work contained data indicating a decrease in the ratio of *Bacteroidota*/*Firmicutes* in the gut and the existence of a correlation between the severity of autism and the severity of gastrointestinal dysfunction.

A similar relationship between dysbiosis and neurological and mental illness was found in the Czech Republic [130]. The results showed altered profiles of the gut microbiome and its metabolites in patients with severe anorexia nervosa, which was primarily reflected in the depletion of the main microbiota. The intestinal composition of the patients was represented by bacteria from the families *Rikenellaceae, Clostridiaceae, Christensenellaceae*, and *Ruminococcaceae*.

Changes in the intestinal profiles of Polish patients with ulcerative colitis were shown [131]. It was found that bacteria of the *Clostridia* and *Bacteroidia* classes are involved in inflammation of the mucous membranes. Increased contents of *Proteobacteria* and *Actinobacteria* were revealed, while the contents of *Bacteroidota* and *Verrucomicrobia* were reduced in comparison with the control group. Similar studies were carried out in the Czech Republic [132]. It was demonstrated that, in patients with ulcerative colitis, bacteria such as *Akkermansia muciniphila, Butyricicoccus pullicaecorum*, and *Clostridium colinum* were decreased in the gut. In studies on the microbiomes of the population of people living in the central part of Russia, an increase in the bacteria *Haemophilus, Olsenella, Prevotella, Cedecea, Peptostreptococcus, Faecalibacterium, Lachnospira, Yersinia*, and *Leuconostoc* was shown [133].

In addition to obesity, cardiovascular diseases, particularly atherosclerosis, are common and dangerous diseases in Europe [108,134]. Bacterial diversity is related to the relationship between intrinsic and environmental factors that are unique to each person. This uniqueness explains the propensity of some people to develop cardiovascular diseases [117]. It was proposed that researchers should consider changes in the composition of the intestinal microbiome as biomarkers for the development of atherosclerosis. The authors considered members of the genera *Prevotella, Bacteroides*, *Clostridium*, and *Faecalibacterium* as such indicators [134]. Monozygotic twins were the objects of study by Hungarian scientists. The control group showed an increased content of the *Prevotellaceae* family compared to patients with subclinical atherosclerosis.

It is known that a pathological immune response can be initiated in the intestine and that it can cause the development of multiple sclerosis [135]. This disease suppresses the normal gut microbiome, a result of which is the onset of a prevalence of pro-inflammatory species within it. The relationship between microbiome-related changes and systemic inflammatory response syndrome was demonstrated in the Hungarian population [136]. This disease is reflected by a decrease in genera such as *Bacteroides, Bifidobacterium Veillonella*, and *Lactobacillus* in the intestine, while the number of *Staphylococcus* and *Pseudomonas* is increased significantly.

A team of scientists from the University of Bratislava examined the microbiomes of patients with inflammatory bowel disease [116]. Intestinal inflammation can alter the balanced microbial community represented by *Bacteroidota, Firmicutes*, and *Actinobacteria*, accompanied by an increase in *Proteobacteria*, including the prevailing members of the family *Enterobacteriaceae*. For example, it was shown that, during the period from June to the end of November, there was a decrease in the bacteria typical of inflammation, such as *Eggerthella lenta*, *Fusobacterium* spp., *Bacteroides* spp., *Helicobacter* spp., etc. The numbers of *Pediococcus* spp., *Clostridium* spp., and *Escherichia*/*Shigella* spp. increased.

In a study of the microbiome, the state of the intestines of patients of different age groups, including children, was assessed. The intestinal condition of children with stable idiopathic nephrotic syndrome was characterized by a decreased *Bifidobacterium* content and an increased *Candida* content in contrast to a healthy group of children [115]. Changes in the composition of the intestinal microbiome were studied in a group of Hungarian children from 3 to 6 years old who were taking probiotics. It was found that the number of *Bifidobacterium* and *Lactobacillus* increased compared to those children whose diet was not changed [114].

It is not only the cases of changes in the microbiome under the influence of a particular disease but also cases of changes under the influence of other factors, such as lifestyle, that attract the interests of researchers. For example, an interesting study examining the gut microbiome in athletes was carried out in Poland [110]. In contrast to the control group, the intestines of the athletes were characterized by a reduced abundance of the main genus of the intestinal microbiota, *Bacteroides*, and a higher abundance of *Prevotella*. The authors explained this finding by the fact that the inverse correlation of the *Prevotella* bacteria was associated with the consumption of sucrose.

The microbiota of the Russian population is of particular interest to researchers since Russia includes a wide range of environmental conditions and ethno-geographic cohorts. In one of the studies, the geography of the sampling covered a significant part of the densely populated zone of the territory of Russia, including regions of Europe and southern Siberia. The urban-type settlements were represented by four of the ten most populous cities in Russia (St. Petersburg, Saratov, Rostov-on-Don, and Novosibirsk), and the rural centers by eleven villages and towns in Tatarstan, Omsk, Tyva, and Khakassia. New features of the Russian microbiomes included gut microbial communities (based on the genera of the phyla *Firmicutes* and *Actinobacteria*, which are associated with a healthy gut) and underrepresented communities rich in *Bacteroidota* [137]. Thus, the Russian microbiome is based mainly on bacteria from the phyla *Firmicutes* and *Actinobacteria*, which nutritionally specialize in starch [138]. Some of the representative species in these groups (*R. bromii* and *E. rectale*) showed an increase when resistant starch was introduced into the diet [139]. The specific traits of the gut microbiota of Russians are likely associated with the hosts’ diet, cultural habits, and socioeconomic status. Presumably, new communities are supported by the high consumption of starch-rich bread and potatoes, which are typical staple foods in Russia [137,140,141].

Another large-scale study also showed significant differences between the microbiomes of the populations of Russia, the United States, Denmark, and China. Most genera of the Russian microbiome belonged to *Firmicutes*, with the presence of *Bacteroidota*, *Verrucomicrobia*, *Actinobacteria*, *Proteobacteria*, *Tenericutes*, and *Archaea kingdom*. Interestingly, new structures of the microbial communities were found to be 2.6 times less frequent in urban hosts than in rural hosts, which suggests that further study of rural microbiota will show an even greater variety of microbiota configurations. In addition, other original structures were identified that were absent in the non-Russian metagenomes. *Akkermansia muciniphila* and *Methanobrevibacter smithii* predominated in two samples [131,142]. The dominance of *Methanobrevibacter* most likely reflects the high hydrogen levels provided by the fermentative bacteria. This seems logical due to the lack of competition with sulfate-reducing bacteria, as zero values for *Desulfovibrio* and *Desulfitobacterium* were observed [143]. Other unique pairs included *Phascolarctobacterium* and *Lactobacillus*, which constituted the most numerous genera in the only rural sample (from the rural Omsk region and Khakassia, respectively). The subgroups of Tatarstan and Tuva differed in their composition; both were formed by similar metagenomes based on butyrate-producing *Firmicutes* (*Agathobacter rectal*, *Eubacterium rectale*, *Coprococcus eutactus*, *Faecalibacterium prausnitzii*, and *Ruminococcus bromii*), while the latter also contained high proportions of the *Firmicutes*. The rural subtypes found were especially interesting because of the strong associations of these bacteria with the health of the host. It was found that the composition of the microbiota profiles of the rural population was similar within each region and was represented by the taxa of bacteria associated with a “healthy” gut. The composition of the microbiota profile in the urban population was less diverse than in the rural population. This difference is most likely associated with the diets of the people [142].

Since some of the identified communities are mainly composed of species associated with a healthy gut, microbiome research is of significant interest for identifying local configurations of the human microbiota and altering the microbiota functions regarding to health and disease.

Moreover, studies of the intestinal microbiomes of the Russian population were carried out about various pathologies. The intestinal microbiota of people suffering from bronchial asthma is characterized by the same diverse taxonomic composition of metagenomes as the microbiota of healthy people; however, some differences were found. In patients with bronchial asthma, an increase in the proportion of *Proteobacteria* was found, due to the prevalence of the classes *Betaproteobacteria* and *Gammaproteobacteria* in allergic asthma, and in patients with the non-allergic phenotype of the disease, mainly due to the class *Gammaproteobacteria*. In bronchial asthma, a disturbance of the metabolic function of the intestinal microbiome was revealed: the proportion of taxa producing butyrate (*Anaerostipes*, *Faecalibacterium*) and acetate (*Alistipes*) decreases [144].

In children with respiratory tuberculosis, the intestinal microbiota has some characteristic features. *Lactobacilli*, which form the basis of a normal intestinal microbiome, were detected in only 53.3% of the examined children. The most pronounced dysbiotic manifestations in adult patients with tuberculosis were those of *Enterococcus* spp. and *E. coli*, with normal enzymatic activity [145].

The gut microbiota largely shapes human health by contributing to the development of various autoimmune diseases, both intestinal and non-intestinal, including multiple sclerosis. Altered composition of gut bacteria was found in patients with primary progressive multiple sclerosis compared to healthy subjects. A marked increase in the relative abundance of the two dominant OTUs (operational taxonomic units), *Gemmiger* sp. And unclassified *Ruminococcaceae* was associated with this disease. The most significant change associated with multiple sclerosis was a relative abundance of some members of the phylum *Firmicutes*, such as *Acidaminococcaceae*, *Eubacteriaceae*, and *Christensenellaceae*, as well as some unclassified *Firmicutes* and *Clostridia*. The family *Desulfovibrionaceae*, with the genera *Bilophila* and *Desulfovibrio*, represented by one OTU of *Bilophila wadsworthia* and seven OTUs of unclassified *Desulfovibrio*, turned out to be more numerous in the cohort with primary progressive multiple sclerosis. Although *Desulfovibrio piger* is found in the intestines of some healthy individuals, higher numbers of this species may be associated with certain gastrointestinal diseases, such as inflammatory bowel disease or autism [146].

The proportion of *Firmicutes* sequences, which are the dominant component of the gut microbiota of healthy people in Russia, was lower in patients with ulcerative colitis, while the proportion of *Proteobacteria* sequences was higher. In some samples, significant sequence contents of the pathogenic representatives of *Firmicutes* and *Proteobacteria* were also found, including *Acinetobacter* spp., *Enterococcus* spp., *K. pneumoniae*, *P. mirabilis*, *S. aureus*, *St. maltophilia*, *Streptococcus* spp., and *C. difficile* [147].

In Russian patients with cholelithiasis, a wide range of bacteria localized in the gallbladder were revealed, mainly representatives of the genera *Bifidobacterium* spp., *Bacteroides* spp., and *Lactobacillus* spp. In addition, a large number of *E. coli*, including the enteropathogenic strains, was detected in the bile of the patients. *Enterococcus* isolated from the gallbladder of patients with cholelithiasis was characterized by a wide range of pathogenicity genes. Moreover, in the microbiocenosis of the gallbladder, a strain of *E. faecium* with a gene providing resistance to the antibiotic reserve vancomycin was found [148].

## 6. Conclusions

All races and nationalities are distinguished not only by their genetic potentials but also by the living conditions in which they are formed. The concept of the multifactorial action of the environment provides a special contribution to the process of the formation, growth, and development of gut microbiota. These factors are, first of all, the geography of residence, social status of a person, religion, health, and socio-cultural environment, as well as food habits and preferences. These factors leave an imprint on the composition of the bacteria inhabiting the gut and their numbers. Therefore, we can discuss a direct relationship between the gut microbiome and ethnicity.

The dominant bacterial taxa of the intestines of any healthy person are *Firmicutes, Bacteroidota*, *Actinobacteria, Proteobacteria*, *Fusobacteria*, and *Verrucomicrobia*, with *Firmicutes* and *Bacteroidota* dominating (90%). To date, several studies have been carried out that can provide generalized information about the features of the intestinal microbiomes of people living in different countries and with different income levels, cultural and religious statuses, dietary habits, and diseases. Thus, the *Prevotella* enterotype of the intestinal microbiome is characteristic of Buddhists and Muslims, while the rest of the representatives of religious movements, including the peasantry, have the *Bacteroides* enterotype. It is important to understand the lifestyle of a person. For example, among athletes, unlike sedentary people, the bacterial taxon *Prevotella* predominates in the gut microbiome. In terms of disease, the dominance of *Firmicutes* over *Bacteroidota* is a characteristic pattern of the gut microbiomes of obese people.

The uniqueness of the healthy intestinal microbiome of a European (including Slavic nationality) lies in the dominance of the phyla *Firmicutes, Actinobacteria, Bacteroidota, Proteobacteria, Fusobacteria*, and *Verrucomicrobia* over the rest of the bacterial taxa, while only *Firmicutes* predominate in the guts of Americans. Among the African population, the characteristic microbiota is *Bacteroides* and *Prevotella*. The gut microbiomes of Asian residents are very diverse and rich in members of the genera *Prevotella, Bacteroides, Lactobacillus*, *Faecalibacterium*, *Ruminococcus*, *Subdoligranulum*, *Coprococcus*, *Collinsella*, *Megasphaera*, *Bifidobacterium*, and *Phascolarctobacterium*. In general, it can be assumed that the Asian type of gut microbiome is the healthiest. This can partly be explained by the food preferences of the inhabitants of this region. Regarding the healthiest microbiome in terms of religion, it is difficult to draw any conclusions about the healthiest microbiome due to the limited publications on this issue.

Further detailed large-scale studies of the bacterial diversity of the intestines of various peoples and races will provide clear information about the distinctive and unique components of the microbiomes inherent in each of them.

## Data Availability

Data supporting this study are available in cited articles where they were originally reported.
